# Tuberculosis of the spermatic cord extended to the testis in a child mimics a testicular tumor: A case report

**DOI:** 10.1016/j.eucr.2025.103056

**Published:** 2025-05-01

**Authors:** Lohourou Grah Franck, Bénié Adoubs Célestin, Traoré Ibrahim, Kpangni Ahua Jean Bertrand, Kobenan Attaa Ange Rebecca, Nandiolo Koné Rose

**Affiliations:** a Pediatric Surgery Unit of the Bouaké Teaching Hospital University Alassane Ouattara, Bouaké, Côte d'Ivoire; b Department of Anatomy Pathology of the Bouake Teaching Hospital, University Alassane Ouattara, Bouaké, Côte d'Ivoire; c Pediatric Surgery Unit of St. Joseph Moscati Catholic Hospital of Yamoussoukro, University Alassane Ouattara, Bouaké, Côte d'Ivoire

**Keywords:** Tuberculosis, Spermatic cord, Testicular

## Abstract

Spermatic cord tuberculosis with testicular involvement is rare and can closely resemble a testicular tumor, particularly in patients with no prior history of tuberculosis. A 5-year-old child presented with a left inguino-scrotal mass of 1 month's duration and underwent left orchiectomy after a presumptive diagnosis of a testicular tumor. Histopathological diagnosis revealed spermatic cord tuberculosis with testicular involvement. The patient followed the tuberculosis management protocol. At 18 months of follow-up, a good clinical outcome was observed. This condition presents a diagnostic dilemma similar to that of a testicular tumor. Orchiectomy can be avoided if frozen section examination is performed.

## 1 Introduction

Tuberculosis of the spermatic cord is rare.^1^^,^^2^ We present a rare case of tuberculosis of the spermatic cord with testicular involvement in a 5-year-old child with the aim of highlighting the similarity of presentation with spermatic cord tumor associated with the testis and the possibility of misdiagnosis which may lead to an unjustified orchidectomy.This manuscript was prepared following the CARE guidelines (https://www.care-statement.org)

## 2 Case report

Child, 05 years old, male, consults for an increase in the volume of his left scrotum. A 5 year old male presented for increase in size of left hemiscrotum.

This painless swelling of the scrotum had been developing for 1 month in an apyretic context. This swelling had gradually increased in size since the beginning. There was no notion of tuberculosis infection. There was no family of TB or known exposure and was not there a travel history.

**Fig. 1:**
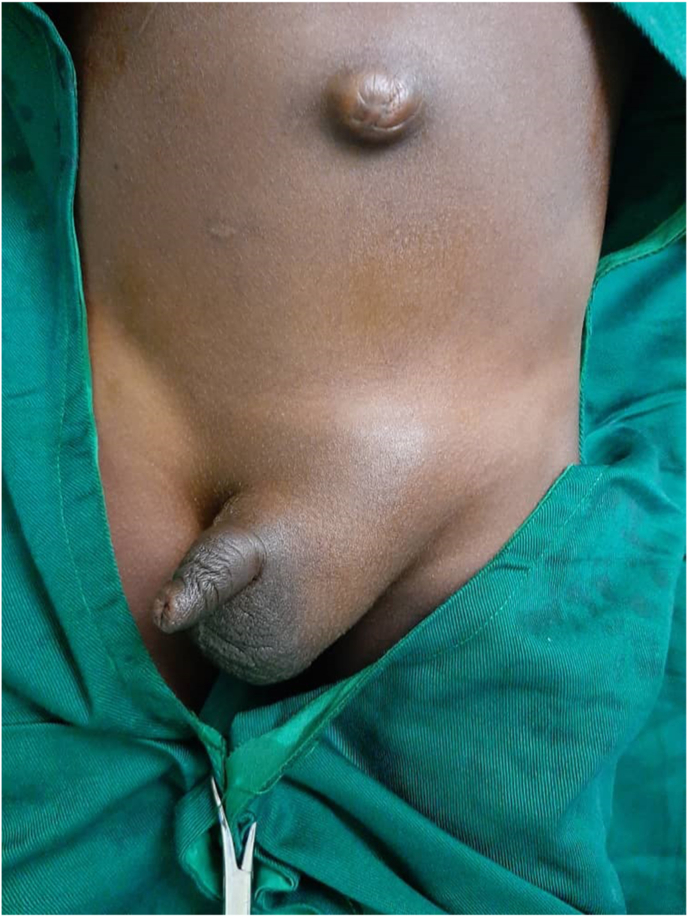
Left inguino-scrotal swelling.

The patient was vaccinated with BCG in childhood without control tuberculin skin test. There was no history of trauma. On clinical examination, the child was in good general condition and in good nutritional status. The left scrotum was increased in volume but without inflammatory features of the skin of the scrotum. Left inguino-scrotal swelling (Fig. 1)

On local examination, the testicle could not be differentiated from the spermatic cord. A hard, painless mass was palpated along the entire inguinal canal to the scrotum. Transillumination test was negative. Examination of the right scrotum was normal with palpation of the spermatic cord and the testicle of normal size. There were no palpable inguinal adenopathy. Abdominal examination was normal. On biological examination: The white blood cell count was 11.9 × 10^9^/L with neutrophils of 31 %; the hemoglobin level was 10.8 g/dl. An inflammatory syndrome with a sedimentation rate (ESR) of 100 mm in the first hour and a C-Reactive protein of 80 mg/L. The dosage of βHCG and α FP was normal. HIV serology was negative.The renal and hepatic assessment (GOT/GPT) was normal. The urine analysis performed revealed no signs of urinary tract infection. Testicular ultrasound revealed the presence of a mass in the left bursa, present throughout the inguinal canal, 40 mm of homogeneous tissue in the major axis, suggesting a testicular tumor.

**Fig. 2:**
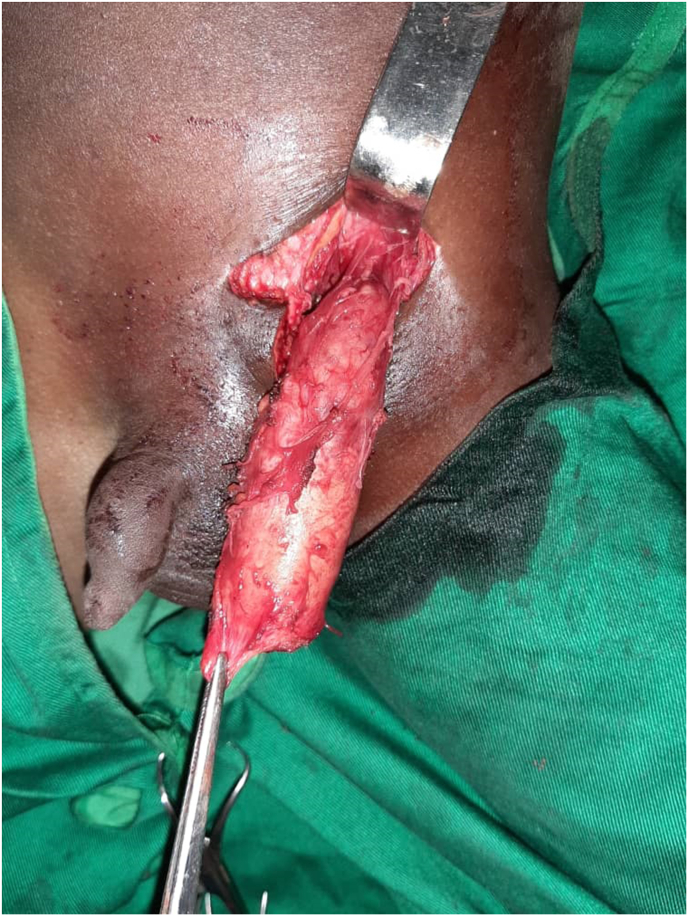
Appearance per operative.

**Fig. 3:**
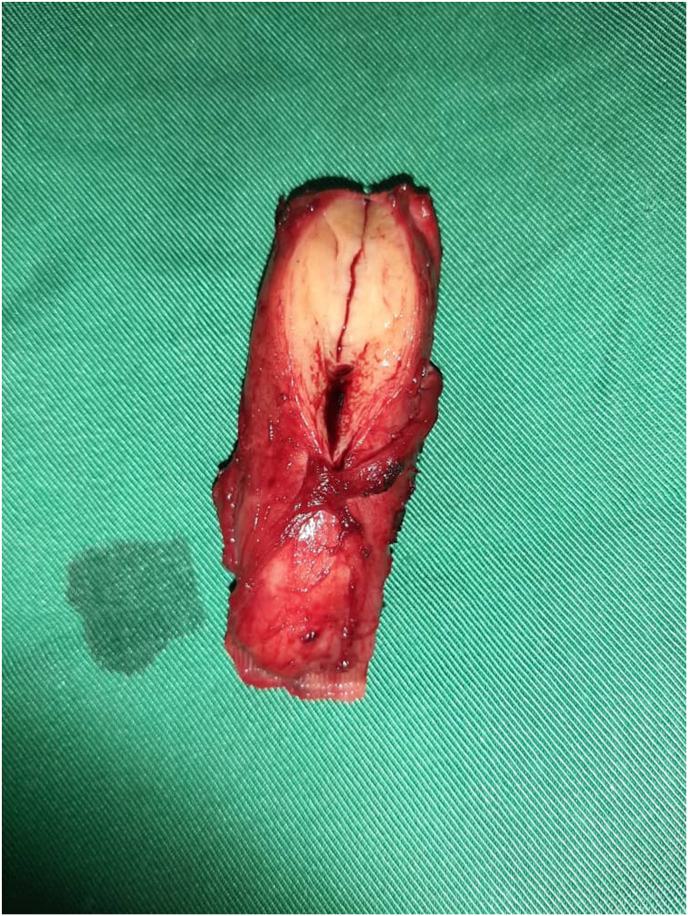
Operative spécimen.

**Fig. 4:**
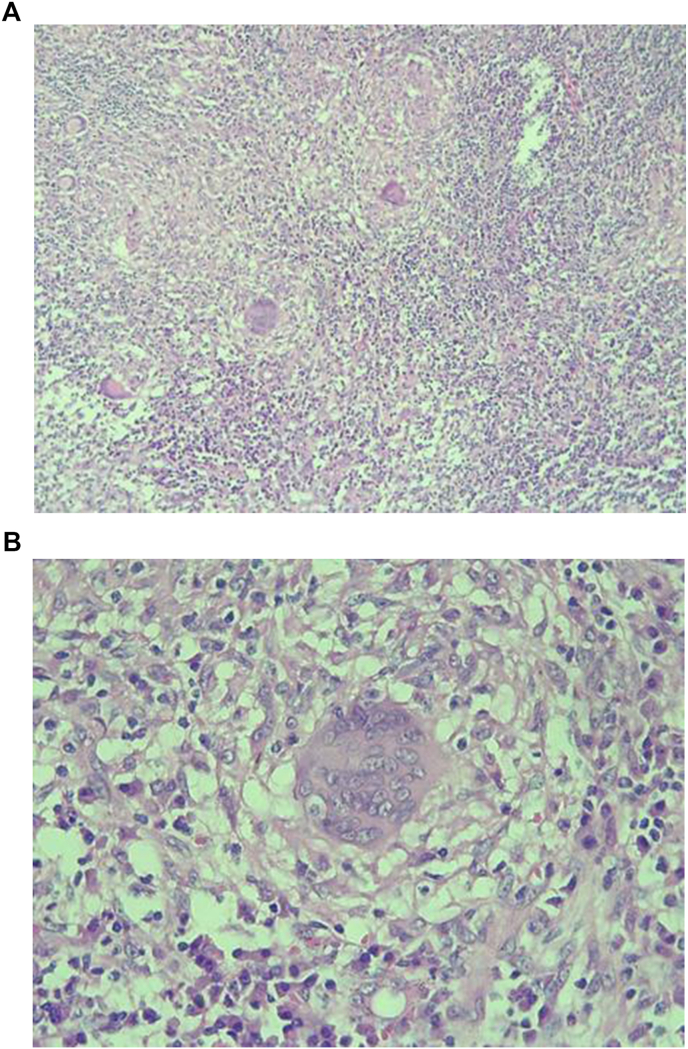
Granulomatous reaction composed of epithelioid cells, Langhans-type giant cells and lymphoplasmocytes. This reaction is centered by a finely granular anhistic caseous necrosis and extends beyond the testis reaching the spermatic cord <˂HE∗10≫ and << HE∗400 ≫

The abdominal ultrasound scan did not reveal any deep lymphadenopathy. A chest X-ray and an abdominopelvic CT scan were normal.We diagnosed a left testicular tumor based on the clinical and ultrasound examination.After obtaining informed and written consent from the parents, we performed a left orchidectomy (intraoperative appearance) (Fig. 2) then the anatomo-pathological examination of the surgical specimen (Fig. 3) reveals the appearance of progressive caseous tuberculosis (Fig. 4). The section planes examined involved testicular tissue that is the site of a granulomatous reaction made up of epithelioid cells, Langhans-type giant cells and lymphoplasmocytes. This reaction is centered by a finely granular anhistic caseous necrosis and extends beyond the testicle reaching the spermatic cord. There is no histological sign of malignancy (Fig. 4). The patient benefited from the tuberculosis management protocol. After 18 months, good clinical progress was noted.

## 3 Discussion

Tuberculosis can affect many organs of the genitourinary region. It is one of the rare forms of extrapulmonary involvement.^3^ Genital tuberculosis is uncommon and testicular-associated spermatic cord tuberculosis is even rarer.^1^^,^^2^ The pathogenesis behind the spread of tubercle bacilli in the spermatic cord is controversial. It can occur secondary to a dormant or active pulmonary source.^4^ In rare cases, contamination by instillation of the bacillus Calmette-Guérin for bladder cancer has also been implicated.^5^ However, for the organisms to reach the spermatic cord, spread occurs either by the hematogenous route or by the vas deferens or lymphatic vessels of the genitourinary tract.^2^ Spermatic cord tuberculosis is a disease that usually affects sexually active men with genitourinary contamination. But the few cases described in childhood suggest the possibility of hematogenous spread of the bacillus. It can also affect patients with pulmonary infection (<1 %).^4^ The main problem with genital tuberculosis lies in the diagnosis, which is often difficult and late in the absence of other suggestive localizations, a notion of contagion or a history of tuberculosis. Indeed, there are no specific clinical signs of genital tuberculosis.^6^ However, it has been observed that most cases of spermatic cord tuberculosis are often misdiagnosed as hernia, cysts, cord lipoma, hydrocele, funiculocele, spermatocele, hematocele, strangulated hernia or omentum, epididymo-orchitis, and tumors of the spermatic cord, rhabdomyosarcoma, testicular, and epididymal, and most of them are usually a postoperative histopathological surprise after patients have undergone unwarranted orchidectomy.^7^^,^^8^

Therefore, to overcome this problem of intervention in clinically unsuspected cases of spermatic tuberculosis, authors have documented that preoperative frozen section can be useful, especially to exclude tumors, thus allowing limited resection of the mass with preservation of the testis and epididymis^.^^9^ Medical treatment is a six-month regimen of anti-tuberculous chemotherapy comprising rifampicin, isoniazid, pyrazinamide, and ethambutol for the first 2 months followed by rifampicin and isoniazid for the next 4 months. However, sometimes, surgical intervention may be necessary.^2^^,^^10^

Moreover, the role of intraoperative frozen section analysis of the testis in sparing unnecessary orchidectomy in case of suspected lesions is feasible and has been studied with favorable results.^11^, ^12^, ^13^

## 4 Conclusion

Tuberculosis of the spermatic cord involving the testis is rare and constitutes a diagnostic dilemma mimicking a testicular tumor. A thorough preoperative workup and frozen section examination when available can avoid unnecessary orchidectomy.

## CRediT authorship contribution statement

**Lohourou Grah Franck:** Writing – review & editing, Writing – original draft, Conceptualization. **Bénié Adoubs Célestin:** Writing – original draft. **Traoré Ibrahim:** Writing – original draft. **Kpangni Ahua Jean Bertrand:** Writing – original draft. **Kobenan Attaa Ange Rebecca:** Writing – original draft. **Nandiolo Koné Rose:** Writing – review & editing.

## Informed consent

Informed consent was obtained from the patient or guardian for publication of this case report and any accompanying images.

## Authorship

All authors attest that they meet the current ICMJE criteria for Authorship.

## Ethical approval

Ethical approval was obtained from the Department of Pediatrics Surgery, University Hospital of Bouaké.

## Funding

There was no funding for this case report.

## Declaration of competing interest

The authors declare that they have no competing interests.

“Generative AI and AI-assisted technologies were NOT used in the preparation of this work”
